# The Cryo-EM Structure of a Complete 30S Translation Initiation Complex from *Escherichia coli*

**DOI:** 10.1371/journal.pbio.1001095

**Published:** 2011-07-05

**Authors:** Patricia Julián, Pohl Milon, Xabier Agirrezabala, Gorka Lasso, David Gil, Marina V. Rodnina, Mikel Valle

**Affiliations:** 1 Structural Biology Unit, Center for Cooperative Research in Biosciences (CIC bioGUNE), Parque Tecnológico de Bizkaia, Derio, Spain; 2 Department of Physical Biochemistry, Max Planck Institute for Biophysical Chemistry, Göttingen, Germany; 3 Department of Biochemistry and Molecular Biology. Faculty of Science and Technology, University of the Basque Country, Bilbao, Spain; Charité - Universitätsmedizin Berlin, Germany

## Abstract

Formation of the 30S initiation complex (30S IC) is an important checkpoint in regulation of gene expression. The selection of mRNA, correct start codon, and the initiator fMet-tRNA^fMet^ requires the presence of three initiation factors (IF1, IF2, IF3) of which IF3 and IF1 control the fidelity of the process, while IF2 recruits fMet-tRNA^fMet^. Here we present a cryo-EM reconstruction of the complete 30S IC, containing mRNA, fMet-tRNA^fMet^, IF1, IF2, and IF3. In the 30S IC, IF2 contacts IF1, the 30S subunit shoulder, and the CCA end of fMet-tRNA^fMet^, which occupies a novel P/I position (P/I1). The N-terminal domain of IF3 contacts the tRNA, whereas the C-terminal domain is bound to the platform of the 30S subunit. Binding of initiation factors and fMet-tRNA^fMet^ induces a rotation of the head relative to the body of the 30S subunit, which is likely to prevail through 50S subunit joining until GTP hydrolysis and dissociation of IF2 take place. The structure provides insights into the mechanism of mRNA selection during translation initiation.

## Author Summary

Translation is the process by which a ribosome converts the sequence of a messenger RNA (mRNA)—produced from a gene—into the sequence of amino acids that comprise a protein. Bacterial ribosomes each have one large and one small subunit: the 50S and 30S subunits. Initiation of translation entails selection of an mRNA, identification of the correct starting point from which to read its code, and engagement of the initial amino acid carrier (tRNA). These events take place in the 30S subunit and require the presence of three initiation factors (IF1, IF2, IF3). Formation of this 30S initiation complex precedes joining with the 50S subunit to assemble the functional ribosome. By using a cryo-electron microscopy approach to visualize the structures without fixation or staining, we have determined the structure of a complete 30S initiation complex and identified the positions and orientations of the tRNA and all three initiation factors. We found that the presence of the initiation factors and tRNA induces rotation of the head relative to the body of the 30S subunit, which may be essential for rapid binding to the 50S subunit and for regulating selection of the mRNA. IF3 had not been seen previously in the context of the 30S structure and its visualization gives insight into a potential role in preventing association of the two ribosomal subunits. These findings are important for understanding how the interplay of elements during the early stages of translation selects the mRNA and regulates formation of functional ribosomes.

## Introduction

Initiation is the most regulated step of translation, at which the ribosome selects mRNAs according to their translation initiation regions (TIR) and establishes a correct reading frame. In bacteria, translation initiation is promoted by three initiation factors (IF), IF1, IF2, and IF3 [1],[2]. The small ribosomal subunit, 30S, is recruited to single-stranded mRNA regions at the TIR [3]. The Shine-Dalgarno sequence (SD) of the mRNA binds to the anti-Shine-Dalgarno sequence (ASD) of 16S rRNA in a cleft between the head and the platform at the back of the 30S subunit, whereas mRNA wraps in a groove that encircles the neck of the 30S subunit [4]–. IFs bind to the 30S subunit and work synergistically to accelerate initiation and ensure the choice of the correct start codon and initiator tRNA. IF1 is a one-domain compact protein which binds at the A site of the 30S subunit [7]. IF2 is a multi-domain GTPase that plays a major role in the recruitment of fMet-tRNA^fMet^ to the 30S IC. The highly conserved C-terminal half of the protein (CTD) contains the GTP- and fMet-tRNA^fMet^-binding domains. The role of the less conserved N-terminus (NTD) is not clear, except that it may provide an additional anchor for IF2 on the 30S subunit [8],[9]. Docking of fMet-tRNA^fMet^ to the complex of the 30S subunit with mRNA and IFs completes the formation of the 30S IC [10]. The initiator tRNA is held in a characteristic position by two interactions: one involving the tRNA decoding stem which is buried in the P site of the 30S subunit, and the other between IF2 and the tRNA acceptor end [11]. The orientation of fMet-tRNA^fMet^ in the complex differs from the canonical P-site position and was designated as P/I state [12]. IF2 and fMet-tRNA^fMet^ provide a large interaction surface for binding the 50S subunit [11]–[13]. Formation of the 30S IC constitutes an important step for the selection of a favorable TIR. The ability of the ribosome to screen the TIR and to check for the fidelity of codon-anticodon interaction strongly depends on the presence of IF3 [14]. However, a structure of a 30S IC with IF3 is not available.

IF3 consists of two domains, IF3C and IF3N, connected by a linker. The structures of the separate domains are known, but not of the full-length protein. The binding site for IF3 on the ribosome has been examined by a variety of biochemical techniques and by cryo-EM [15]–[18]. Overall, IF3 appears to bind to the platform of the 30S subunit, but the domain orientation of IF3 and exact binding contacts for each domain remain controversial. IF3 has several functions during translation initiation. It interferes with ribosomal subunit association [19], affects the rates of tRNA association to and dissociation from the P site [20],[21], and ensures the fidelity of translation initiation [22]–[24]. Most importantly, IF3 is crucial for sensing the TIR of mRNA. In the absence of IF3, the ribosome is largely unable to discriminate against unfavorable TIRs or incorrect initiation codons [14],[25].

During the last years, structures of the 30S IC and 70S IC in the absence of IF3 [11],[13] and of the 70S IC with density attributable to IF3 [12] have been reported. However, the structure of the complete 30S IC has remained elusive, probably due to intrinsic structural dynamics of the 30S subunits. Comparison of the 70S IC structures in the GTP- and GDP-bound states of IF2 revealed that in the pre-hydrolysis state the 30S subunit is found in a rotated (ratcheted) state relative to the 50S subunit. Consistent with this notion, single molecule FRET data have shown that docking of the 50S subunit to the 30S IC initially forms the 70S IC that is in the rotated conformation, and that GTP hydrolysis by IF2 promotes the rearrangement from the rotated to non-rotated state, thus enabling the ribosome to progress into the elongation phase [26].

Here, we present the cryo-EM reconstruction of the complete 30S IC containing all three IFs, mRNA, and fMet-tRNA^fMet^. We analyze the conformational changes of the 30S subunit induced by binding of IFs and tRNA and identify the position and orientation of IF3 and of the NTD of IF2. Unlike the 30S IC obtained in the absence of IF3 [11], the complete 30S IC studied in the present work reflects a translation initiation intermediate that is fully competent in the selection of the TIR and the correct start codon. The data show a rotation of the head of the 30S subunit upon formation of the 30S IC and suggest how the conformational changes of the 30S subunit could regulate 50S subunit joining and mRNA selection.

## Results

### Reconstruction of a Complete 30S IC

**Figure 1:**
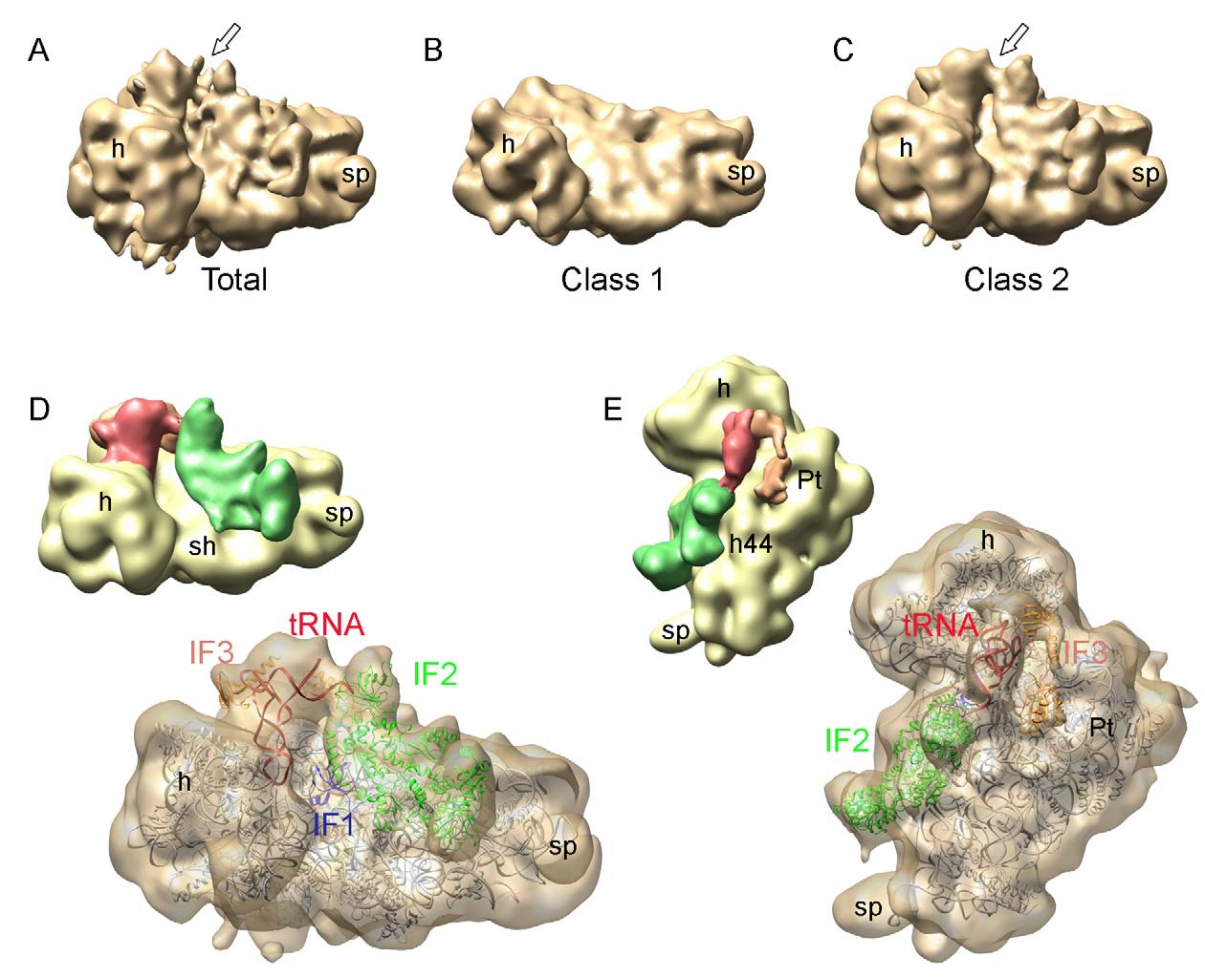
Cryo-EM reconstruction and classification of the 30S IC. (A) Initial reconstruction of the 30S IC with partial densities for tRNA and IF2 on the surface of the 30S subunit (arrow). (B) Cryo-EM map of class 1 particles after ML3D classification of the total set of images. No density for tRNA or IF2 can be seen. (C) The cryo-EM map of class 2 particles showing improved density for tRNA and IF2 on the 30S subunit (arrow). This map was assigned to the 30S IC complex. (D and E) Two orientations of the cryo-EM map for the 30S IC complex after segmentation and fitting of atomic coordinates attributed to the 30S subunit (gray), IF2 (green), IF1 (blue), fMet-tRNA^fMet^ (red), and IF3 (orange). Smaller thumbnails depict segmented map with solid densities in different colors, while large renderings in semi-transparent representation show fitted atomic coordinates. Landmarks on the 30S subunit in all figures indicate: h, head; sp, spur; sh, shoulder; pt, platform; h44, helix 44 from the 16S rRNA.

The 30S IC was prepared from *E. coli* 30S subunits, IF1, IF2, IF3, fMet-tRNA^fMet^, mRNA, and a GTP analog, GDPNP (Materials and Methods). The mRNA used for complex formation was m002, which contains a 9-nt SD and a 5-nt spacer between the SD and the AUG start codon [27]. The extended SD provides strong interactions with the ASD in the 16S rRNA and drastically reduces the dissociation rate of IF3 [14]; m002 is similar to mRNAs with “enhanced” SD used in previous structural and biochemical studies [11]–[14],[26],[28]. An initial set of 48,000 particles resulted in a cryo-EM map that showed density which could be attributed to tRNA and IF2 on the surface of the 30S subunit (Figure 1A) at positions expected from a previous cryo-EM reconstruction of the 30S subunit in complex with initiator tRNA and IF2 [11]. However, the densities in our initial reconstruction did not reveal structural details of the tRNA or IF2, suggesting heterogeneity of the sample due to differences in the occupancy of the 30S subunit with ligands and/or distinct conformational states. Separation into two classes by non-supervised maximum likelihood–based classification (ML3D) [29], a recently developed tool that has been successfully applied to other ribosomal samples [30], and independent image processing yielded the 3D maps shown in Figure 1B and 1C. The EM map for class 1 particles (23% of all particles) showed no density for IFs or tRNA (Figure 1B). Class 2 accounted for the majority (77%) of the particles, and the corresponding 3D map revealed the presence of fMet-tRNA^fMet^ and IFs (Figure 1C). In the latter reconstruction, the putative densities for IF2 and tRNA could be seen at a higher density threshold compared with the map obtained from the total set of images. Structural details of the initiator tRNA from the X-ray structure [31] and of IF2 known from previous cryo-EM reconstructions [11]–[13] were clearly recognizable. Importantly, high-density threshold rendering revealed density for the SD-ASD duplex for both classes (Figure S1), indicating that class 1 represented 30S·mRNA complexes, rather than vacant 30S subunits. The resolutions estimated at 0.5/0.14 cut-off criteria in the Fourier shell correlation (FSC) were 16.8/14 Å (total set of images), 21/17 Å (class 1, representing the 30S-mRNA complex), and 18.3/15 Å (class 2, representing the 30S IC). Classification into more classes or re-classification of class 2 did not result in improvement of resolution or identification of further conformational states. The limited resolution of the reconstructions may reflect inherent flexibility within the 30S IC induced by IF3-IF1 binding to the 30S subunit, as suggested by kinetic experiments [14].

### Segmentation of the Map for the 30S IC

After removing the density corresponding to the 30S subunit from the class 2 map (Figure 1C), positions for tRNAs and IFs could be assigned, which were consistent with previous structural and biochemical studies (Figure 1D and 1E). The main part of the resulting density could be attributed to the IF2·fMet-tRNA^fMet^ complex placed along the shoulder and the cleft between the head and the body of the 30S subunit. The density for IF1 was not obvious within the 30S IC map, because its binding site was covered by the IF2 structure. However, fitting the crystal structure of IF1 bound to the 30S subunit [7] suggested that, in our map, IF1 can be embedded in the boundary between the 30S subunit and IF2 (Figure 1D) in the cleft formed by the 530 loop, helix 44 of 16S RNA, and ribosomal protein S12 [7]. The moderate resolution of the current 3D map and the lack of a complete atomic model for IF2 (see below) precluded an unambiguous definition of the boundary between IF1 and IF2. Additional densities observed close to the initiator tRNA and the platform of the 30S subunit were assigned to IF3 (Figure 1E). Thus, the cryoEM map of the 30S IC had densities attributable to all the elements of the complete complex (see below for further details of fitting).

### The 30S Subunit Head Rotation

**Figure 2:**
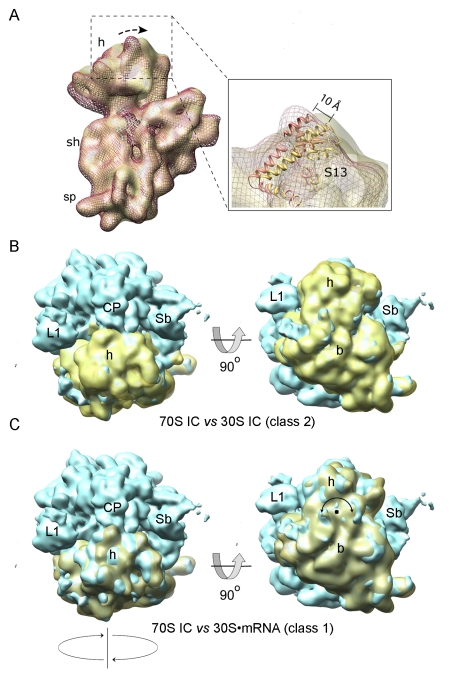
Conformation of the 30S subunit within the 30S IC. (A) 30S subunit conformations in reconstructions of particles from class 1 (red mesh) and class 2 (semitransparent yellow). The arrow indicates the clockwise rotation of the 30S subunit head. The inset shows the positions of protein S13 in the two maps. (B) The current 30S IC map (class 2 after ML3D classification) aligned with the cryoEM map for the 70S IC (EM database code 1249 [12]) in two distinct orientations. (C) Comparison of the 30S·mRNA map (class 1 after classification) with the 70S IC map [12]. The alignment between density maps was performed by maximum overlap of the body of the 30S subunits.

To investigate whether the binding of initiation factors and fMet-tRNA^fMet^ changed the conformation of the 30S subunit, we compared the structures of 30S subunits from classes 1 and 2 (Figure 2). When the maps were superimposed in such a way as to produce a maximal correlation of the 30S bodies, a clockwise rotation of the head toward the E site of the subunit in the 30S IC was found, compared to the 30S·mRNA complex (Figure 2A). Measured by the position of the globular domain of ribosomal protein S13 as a reference point, the displacement was about 10 Å, corresponding to a rotation of the 30S head of approximately 4–5°. The direction and extent of the rotation in the 30S IC map are very similar to those found in the 70S IC (Figure 2B) [12], as opposed to the conformation of the 30S subunit in the map attributed to the 30S·mRNA complex (Figure 2C).

### IF2 Structure and Interactions in the 30S IC

**Figure 3:**
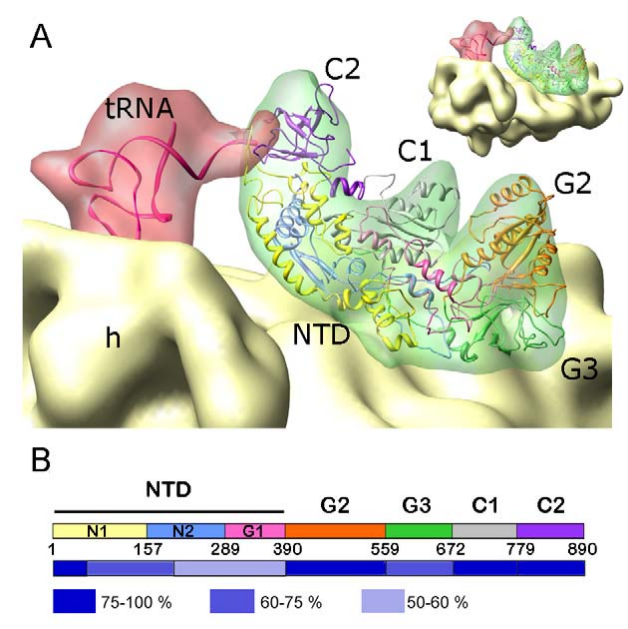
Homology model for IF2 guided by the cryo-EM map. (A) Close-up view of the fMet-tRNA^fMet^·IF2 complex on the 30S IC. Densities for fMet-tRNA^fMet^ and IF2 were rendered semitransparent to show the fitted IF2 (ribbon representation). For fMet-tRNA^fMet^ (pdb code 2FMT; [31]), a rigid-body fitting was performed. Domains G2 (orange), G3 (green), C1 (grey), and C2 (purple) of IF2 are indicated. The NTD was modeled as three sub-domains (yellow, blue, and pink). (B) Domain structure of IF2. Colors are as in (A). Below the segment for each domain, the homology T-Coffee score [72] between IF2 from *E. coli* and the atomic coordinates used is shown.

The crystal structure of full-length *E. coli* IF2 is not available so far. To interpret the density attributed to IF2 and to identify the interactions of IF2 within the 30S IC, a domain homology model of IF2 was constructed (Figure 3). The C-terminal half of the protein (CTD; G2, G3, C1, C2 domains) is highly conserved, whereas the N-terminus (NTD; N1, N2, G1 domains) varies in both amino acid composition and length and is absent in archaea [32]. The NTD is lacking in IF2/eIF5B from *Methanobacterium thermoautotrophicum*, the crystal structure of which is available [33] and which has been used for homology modeling of IF2 from *T. thermophilus* and *E. coli* [11],[12]. To model the full-length *E. coli* IF2, including the NTD which was not visualized previously, we combined several tertiary structures based on sequence homology (Figure S2). Rigid-body fitting was carried out independently for each of the modeled homologous domains. Subsequently, all of them were combined into a single structural model, which was fitted into the density of the 30S IC using MD-based flexible fitting (Materials and Methods). In the MD simulations, secondary structure elements were conserved, and most of the large changes are related to long loops, especially in the NTD region. During the flexible fitting the cross-correlation between atomic coordinates and the EM map improved significantly (from 0.87 to 0.95 estimated at the current resolution). The evolution of the fitting was also monitored by comparison with the initial IF2 model, and in the last cycles the RMSD values between the initial and flexible-fitted models reached a plateau of 8 Å. The overall arrangement of domains in the CTD of the present model of IF2 (Figure 3) is in agreement with the previous models based on cryo-EM [11]–[13], except for some differences that are discussed below. The expected volume of the modeled NTD domains (irrespectively of the uncertainties of their modeled positions) nicely accounted for the observed cryo-EM density indicating the location of the NTD at the 30S subunit surface.

**Figure 4:**
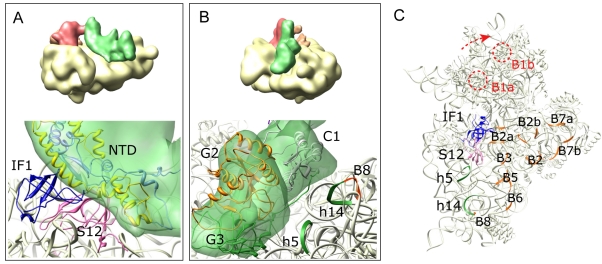
IF2 on the 30S IC. (A) Interactions of the NTD of IF2 with IF1 and S12. Thumbnail shows orientation. (B) Interactions of domains G2 and G3 of IF2 with 16S rRNA. Residues of helix h14 involved in formation of the intersubunit bridge B8 are highlighted in orange. (C) Position of IF2 relative to intersubunit bridges. The binding site for IF2 entails 16S rRNA helices h5 and h14 (green), and proteins IF1 (blue) and S12 (pink). The crystal structure of IF1 bound to the 30S subunit is taken from (pdb code: 1HRO; [7]). Intersubunit bridges are shown in orange. Bridges in the head of the 30S subunit are highlighted in red and the arrow indicates the direction of the 30S subunit head movement.

In the 30S IC, IF2 makes extensive contacts with 16S rRNA (helices h5 and h14), IF1, and S12 (Figure 4). The model suggests that the contacts to IF1 and ribosomal protein S12 are made by the NTD of IF2 (Figure 4A), in line with the ability of the isolated NTD of *E. coli* IF2 to bind to the 30S subunit [9],[34]. Domain G3 of IF2 interacts with helix h5 of 16S rRNA and domain C1 of IF2 with helix h14 (Figure 4B). This set of connections between IF2 and the 30S subunit is similar to the one described in the 70S IC [12], whereas in the 30S IC lacking IF3 the contacts between IF2 and the 30S subunit were seen as small connections involving G3 domain from IF2 and helices h5 and h14 from the 16S rRNA [11]. Interactions with IF1 or S12 were not observed, probably due to the shortened NTD in IF2 from *T. termophilus*. The position of IF2 on the present 30S IC does not interfere with intersubunit bridges formed upon 50S subunit joining (Figure 4C). The only bridge in the vicinity of IF2 is bridge B8, which is established via helix h14 [35]. However, the residues involved in the connection with the 50S subunit (highlighted orange in Figure 4B) are on the face of the helix opposite to the IF2 contact, suggesting that this interaction of IF2 with the 30S IC does not interfere with the binding of the 50S subunit.

### Novel P/I Position of fMet-tRNA^fMet^

**Figure 5:**
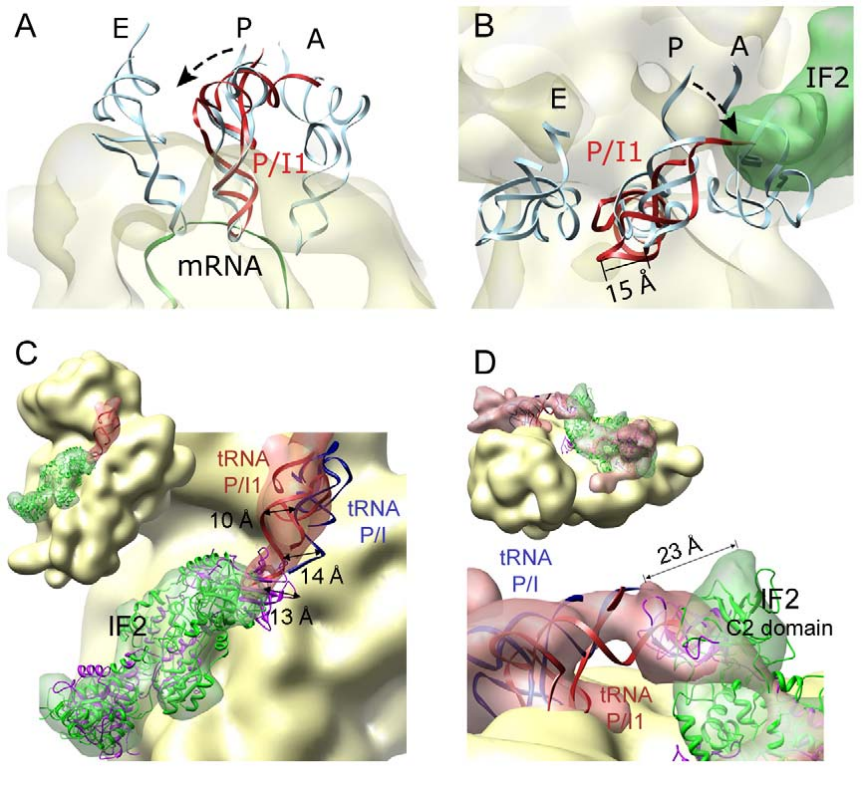
Orientation of fMet-tRNA^fMet^ bound to the 30S IC. (A) Comparison of the positions of fMet-tRNA^fMet^ in the 30S IC (red) with the atomic coordinates for A-, P-, and E-site tRNAs (pdb code: 2HGP; [41]). The arrow indicates a tilt of the fMet-tRNA^fMet^ by around 10 degrees compared to the tRNA in the P site. (B) Top view showing the 15 Å rotation of fMet-tRNA^fMet^ in the complex with IF2 (semitransparent green). (C and D) Comparison between the P/I1 and P/I sites. In (C) densities for IF2 and tRNA from the 30S IC are shown semitransparent, allowing us to visualize fitted coordinates for tRNA (red ribbons) and IF2 (green). For comparison, coordinates fitted in the 70S IC (pdb code: 1ZO1 [12]) are represented for IF2 (purple ribbons) and tRNA in the P/I site (blue). In (D) density for IF2 and tRNA is taken from the 70S IC (EM database code 1249) and depicted semi-transparent. Atomic coordinates are as in (C).

Comparison of the fMet-tRNA^fMet^ position in the present 30S IC reconstruction with crystal structures of ribosomes with tRNAs in A, P, and E sites (Figure 5) reveals that the anticodon loop of fMet-tRNA^fMet^ is positioned essentially in the P site of the 30S subunit, while the tRNA elbow in the 30S IC is tilted approximately 10° towards the E site (Figure 5A). The tilt is similar to that in the P/I intermediate position for the initiator tRNA previously described in cryo-EM studies of the 30S IC and 70S IC [11],[12]. However, unlike the P/I state visualized before, where the acceptor arm of the tRNA was shifted towards the E site [11],[12], the CCA end of fMet-tRNA^fMet^ in our reconstruction is oriented towards the A site by its interaction with the C2 domain of IF2 (Figure 5B). The differences in the orientation are clearly seen at the junction between fMet-tRNA^fMet^ and the C2 domain of IF2 in the respective complexes (Figures 5C,D and S3). Notably, the present 30S IC and the 70S IC reported by [12] have been prepared in the presence of all three IFs and are from the same organism and yet the observed P/I positions are clearly distinct (Figure 5C,D). To distinguish between the two states of the initiator tRNA, we name the state observed in our structure P/I1. Compared to the P-site tRNA, the P/I1 position requires a rotation of the acceptor stem of the tRNA of around 15° (Figure 5).

### Location of IF3 in the 30S IC

**Figure 6:**
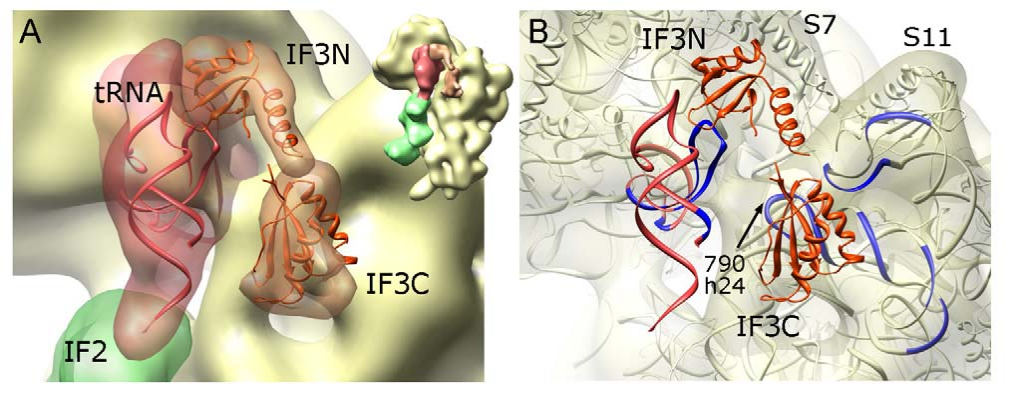
IF3 on the 30S IC. (A) Density for IF3 (semitransparent orange) fitted using the atomic coordinates for IF3 domains, IF3N (pdb code: 1TIF; [36]) and IF3C (pdb code: 2IFE; [37]). Thumbnail shows the orientation. (B) Same orientation as in (A) with semitransparent 30S subunit. Regions on 16S rRNA and tRNA in the proximity of IF3, based on hydroxyl radical footprinting [17], are shown in navy blue. Arrow indicates helix h24 and the loop around nucleotide 790 in 16S rRNA.

Apart from the density for the fMet-tRNA^fMet^·IF2 complex, a bilobed density was observed connecting the 30S platform and the elbow region of the fMet-tRNA^fMet^ (Figure 1E). Subtracting the volume occupied by the 30S·fMet-tRNA^fMet^·IF2 complex from the 30S IC reveals a density comprising two domains connected by a linker. According to its size and position, the density was attributable to IF3 (Figure 6). Rigid-body fitting of the atomic coordinates of the N- (IF3N) and C-terminal (IF3C) globular domains of IF3 from *Geobacillus stearothermophilus* [36] linked by an α-helix yielded a good fit of the EM map. For detailed fitting, IF3C from *E. coli* was used [37]. The density for IF3 in our cryo-EM map had to be visualized at a lower threshold compared to that of tRNA·IF2 (Figure S4), presumably due to incomplete occupancy of the 30S IC with IF3. Further sorting of the class 2 particles did not separate distinct conformational/occupancy states. The two domains of IF3 were placed in such a way that IF3N contacted the elbow region of fMet-tRNA^fMet^, whereas IF3C was engaged in interactions with the 30S subunit. Cross-correlation measurements support the assignment of IF3 domains, since the alternative arrangement after domain swapping reduces the coefficient from 0.79 to 0.65. IF3C was bound to the 790 loop of h24 of 16S rRNA in the vicinity of fMet-tRNA^fMet^; however, no direct contact between IF3C and tRNA was found (Figure 6B).

### Implications for Subunit Joining

**Figure 7:**
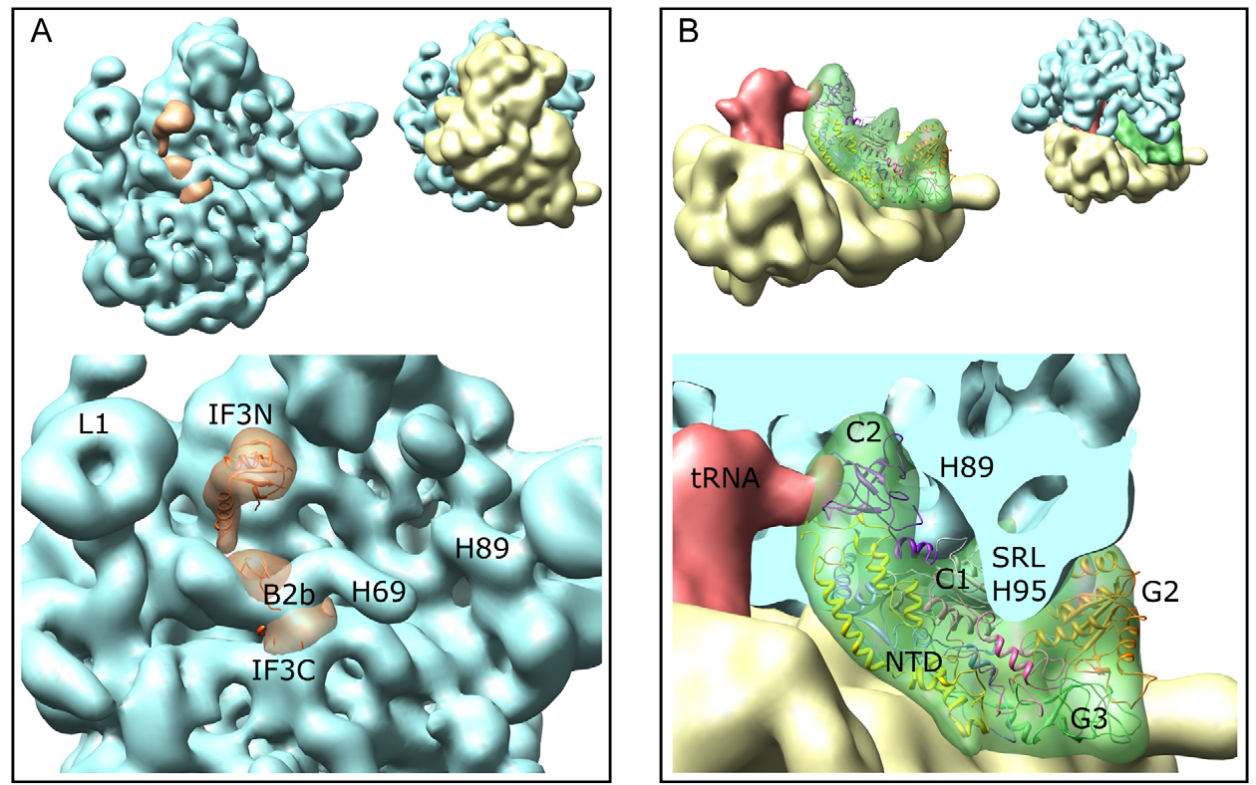
Docking of the 50S subunit onto the 30S IC. (A) Steric clash between IF3C and helix H69 of 23S rRNA at intersubunit bridge B2b. The present map for the 30S IC was aligned with the 70S IC [12]. (B) IF2 as an important determinant for the 50S subunit association. The position of the sarcin-ricin loop (SRL) at the cleft formed by domains G2 and C1 of IF2 is indicated. In both panels, the thumbnails show the 30S IC docked onto the 50S subunit. Labels on the 50S subunit indicate helices in 23S rRNA (H69, H89, and H95) and the stalk region for protein L1.

To examine whether the conformation of the 30S IC was suitable for 50S subunit joining, we aligned our 30S IC map with that of the 70S IC [12]. The position for IF3C in the 30S IC would impair the formation of bridge B2b at the interface between the ribosomal subunits (Figure 7A), suggesting that in this arrangement IF3 in the complex physically impairs subunit joining. The fMet-tRNA^fMet^·IF2 complex provided an extensive surface area for the interaction with the 50S subunit (Figure 7B). Binding of the 50S subunit would position the junction between IF2 and the CCA end of fMet-tRNA^fMet^ close to the peptidyl transferase center. The region containing the sarcin-ricin loop (SRL) of 23S rRNA docked accurately in a cleft formed by domains G2 and C1 of IF2. The GTPase domain, G2, is oriented toward the SRL in the same way as domain I of EF-Tu [38]; the contact with the SRL is expected to be important for the GTPase activation of IF2 [39].

## Discussion

### Structure of the 30S IC

In this study we present the cryo-EM reconstructions of the complete *E. coli* 30S IC containing initiator tRNA, mRNA, and all three initiator factors, as well as of the 30S subunit in the complex with the mRNA alone. The comparison of the 30S IC and the 30S·mRNA complex revealed that the 30S subunits were present in different configurations in the two complexes. In the 30S IC, the head of the 30S subunit was rotated with respect to the body, which can be attributed to the presence of IFs and fMet-tRNA^fMet^. Early site-directed crosslinking studies suggested that the formation of the SD-ASD complex in the absence of IFs places the mRNA in a “standby” position, from which it is shifted backward, closer to the P site upon binding of initiation factors, in particular IF3, before the interaction with the large subunit takes place [40]. Similarly, crystal structures of the 70S complexes indicated that mRNA moves in the 3′→5′ direction with simultaneous clockwise rotation and lengthening of the SD duplex, bringing it into contact with ribosomal protein S2 [41]. The conformational change in the 30S IC induced by factor binding moves the head of the subunit in the right direction along the pathway of the mRNA and could promote the reported back-tracking of the messenger. At the current resolution, the conformation of the 30S subunit in the 30S IC is the same as in the 70S IC before GTP hydrolysis by IF2, where the 30S subunit is found in a rotated (ratcheted) orientation with respect to the 50S subunit [12] when compared with a 70S post-initiation complex [42]; it should be noted that the origin of initiation components (*E. coli*) and the mRNAs (extended SD sequence) are very similar in the present study and in [12], allowing for such detailed comparisons. The change in the 30S configuration is similar to that described within other 70S complexes along several steps of translation (see Figure S5) [30],[43]–[52], but in the current study, the 30S conformation does not require the presence of the 50S subunit. Conformational changes of the 30S subunit were not described in the 30S initiation complex lacking IF3 [11]. This suggests that binding of IFs, in particular IF3, could induce or stabilize the altered 30S subunit conformation. This conformational state of the 30S subunit appears to be retained upon 50S subunit joining until GTP hydrolysis and dissociation of IF2 [26].

The positions of fMet-tRNA^fMet^ and IF2 in the complete 30S IC are similar to, but not identical with, those found in the previously reported reconstructions. As in all available structures, the anticodon stem of the tRNA is buried in the P site of the 30S subunit. However, the position of the tRNA CCA end differs in the reported P/I states. Comparing the 30S IC with the 70S IC from *E. coli* (both with GDPNP) suggests that, upon binding of the 50S subunit, the orientation of the CCA end of the tRNA changes from the P/I1 state, pointing towards the A site of the peptidyl transferase center (30S IC; this article), towards the P/I state, which resides between the P and E sites (70S IC; [12]). The position of the C2 domain of IF2 to which the fMet moiety of fMet-tRNA^fMet^ is bound changes accordingly. A somewhat different P/I state was reported for the 30S IC from *T. thermophilus* formed with GTP in the absence of IF3 [11], which may reflect the known effect of IF3 on the stability of the tRNA binding to the 30S IC. Alternatively, different P/I states may reflect the flexibility of the CCA end of the tRNA. It is possible that all described P/I positions can be sampled during the transition of the initiator tRNA toward the final P site and are important for discrimination of mRNAs with unfavorable TIR (see below).

The density in the cryo-EM map accounted for the entire IF2 and allowed us to map the domain contacts of the factor. As expected, the C2 domain of IF2 binds fMet-tRNA^fMet^ at the single-stranded acceptor end and the fMet moiety of fMet-tRNA^fMet^ [53]. Kinetic studies suggested that IF2 binds to the 30S subunit independent of the tRNA and recruits fMet-tRNA^fMet^ to the 30S IC [10]. The NTD of IF2 contacts IF1 and S12, the latter interaction in line with the biochemical evidence suggesting that the isolated NTD of *E. coli* IF2 can bind to the 30S subunit [9],[34]. The ribosome-bound IF2 provides a large surface area for the joining of the 50S subunit, which would dock in a correct position for the activation of GTP hydrolysis in IF2.

IF1 binds at the A site of the 30S subunit [7]. On the ribosome, IF1 from *E. coli* interacts with IF2, stabilizes IF2 binding [9], and accelerates IF2-dependent fMet-tRNA^fMet^ recruitment [1],[2]. The present results suggest that the stimulatory effect of IF1 may be mediated by a direct contact with the NTD of IF2. In contrast, thermophilic IF1 does not interact with the NTD of IF2 and does not augment IF2 functions [54]; consistently, no direct contact between IF1 and IF2 was found in the *T. thermophilus* 30S IC reconstruction [11]. The NTD region is significantly shorter in IF2 from *T. thermophilus* compared to *E. coli*, suggesting that the IF1–IF2 interaction is not universally conserved [54].

IF3 binds simultaneously to the fMet-tRNA^fMet^ via IF3N and to the 30S subunit at the 790 loop of 16S rRNA via IF3C. The position of IF3C is consistent with hydroxyl radical probing data [17] which located the IF3C binding site close to helices h23, h24, and h45 at the 30S platform in the vicinity of the P site (Figure 5B). Mutation of nucleotide 791 of h24 resulted in a 10-fold decrease of the affinity for IF3 [55]. The 790 loop of h24, which by hydroxyl radical footprinting was located in the vicinity of IF3C [17], was found in contact with IF3C in our reconstruction. However, IF3N in the present complex assumes an orientation that differs from previous models and contacts the elbow region of fMet-tRNA^fMet^. Notably, most of the footprinting probes from IF3N failed to cleave 16S rRNA, making the previous placement of the N domain uncertain [16],[17]. In the cryo-EM reconstruction of the 70S IC [12], an extra density at the platform of the 30S subunit was attributed to IF3. Although no detailed modeling was carried out in that work, the position of IF3N, contacting the elbow region of the initiator tRNA, appears similar to our 30S IC. The position of IF3C in the 70S IC, filling the space between helix H69 of the 50S subunit and initiator tRNA and contacting the anticodon arm of fMet-tRNA^fMet^, seems shifted compared to that in the present 30S IC reconstruction, consistent with the necessity to remove IF3C from the binding site of bridge B2b. The different IF3 positions in the 30S IC and 70S IC may reflect the rearrangement of IF3C upon binding of the 50S subunit; further structural work on the 70S IC complexes will be necessary to substantiate this notion.

### Implications for mRNA Selection

In *E. coli*, mRNAs typically contain a SD sequence of 5 nt or less and a 5–9 nt spacer between the SD sequence and the initiation codon [56]. Variations within the SD region or in the distance between the SD sequence and the start codon strongly influence the efficiency of translation [57],[58]. Kinetic evidence suggested that the regulation occurs at the step of the conversion of the 30S IC into the translating 70S IC, i.e. 50S subunit joining and dissociation of IF3 and IF1 [14], and that the ribosomes discriminate against an mRNA with a strong SD sequence and a short spacer to the start codon, such as the one used in this study. The present structure suggests several potential mechanisms by which the rate of 50S subunit joining may be regulated.

One very likely reason is the positioning of IF3C, which hinders the formation of the intersubunit bridge B2b to the 50S subunit. In this case, the rate-limiting step for 50S subunit association and IF3 release observed in kinetic experiments [14] may reflect an IF3 rearrangement—for example, the movement of IF3C away from the 790 loop of 16S rRNA, which would allow the bridge to form. Another possible mechanism for tuning 50S subunit joining is the orientation of the 30S subunit head relative to the body, which is rotated in the complex with IF3, but not in the 30S complex without IF3 [11]. It is conceivable that the relative movement of the head of the 30S subunit alters the formation of bridges during 50S subunit joining, even though the body of the 30S subunit, IF2, and initiator tRNA provide multiple docking interactions. Consistent with this notion, kinetic experiments suggest that the omission of IF3 restores the rapid 50S subunit joining even for 30S IC with an extended SD sequence [14]. Yet another reason for slow 50S subunit joining may be the particular orientation of IF2 and fMet-tRNA^fMet^ observed in the present 30S IC compared to the complex without IF3 [11] on an mRNA with extended SD sequence; both orientations would be compatible with 50S subunit joining, but one of them might be more favorable. In this case, IF3 and IF1 may affect the positions of fMet-tRNA^fMet^ and IF2 through their respective direct contacts. Apparently, a strong SD-ASD interaction stabilizes the 30S IC in the given conformation, which is maintained through 50S subunit joining [12] and the dissociation of IF1 and IF3 [13], and relaxes only after GTP hydrolysis and dissociation of IF2 [26]. While most of the structural and functional data published so far pertain to mRNAs with a very strong SD [11]–[13],[26],[59], it would be important in the future to obtain structures of initiation complexes with other, more physiological mRNAs.

## Materials and Methods

### Preparation of the 30S IC

30S subunits from *E. coli*, IFs, and fMet-tRNA^fMet^ were prepared as described [14]. 30S subunits (0.1 µM), IF1 (0.3 µM), IF2α (0.2 µM), IF3 (0.3 µM), 002 mRNA (0.6 µM), fMet-tRNA^fMet^ (0.6 µM), and GDPNP (0.5 mM) were incubated at 37°C for 15 min in buffer A (50 mM Tris-HCl, pH 7.5, 70 mM NH_4_Cl, 30 mM KCl, and 7 mM MgCl_2_). Immediately before grid preparation and vitrification, the mixture was diluted to 30 nM 30S IC with buffer A containing 0.5 mM GDPNP.

### Cryo-EM, Image Processing, and Image Classification

Thin carbon was floated onto Quantifoil grids (Quantifoil Micro Tools GMBH, Jena, Germany). A 3.5-µl aliquot of the sample was placed on each grid. Grids were blotted, plunge-frozen in liquid ethane, and stored in liquid nitrogen until data collection. Low-dose images were taken on Kodak SO-163 films in a JEM-2200FS electron microscope (JEOL) operated at 200 kV at a magnification of 50,000. Micrographs were scanned on a Z/I Photoscan scanner (Zeiss) with a step size of 14 µm, resulting in a final pixel size of 2.82 Å. A collection of 238 micrographs was assigned to one of 28 defocus groups ranging from 0.6 to 4 µm underfocus. The 3D reconstruction for the total set of images followed reference-based projection matching in Spire-Spider package [60]. The final resolution was determined by using the Fourier-shell correlation curve with a 0.5 cut-off. Non-supervised maximum-likelihood classification (ML3D) of the images was used from the Xmipp package [29],[61]. Based on the maxima of the probability functions upon convergence of the likelihood optimization, the dataset was separated into two groups. The data accounting for each group were further refined separately, following the same procedure as described for the total number of particles. Rigid-body fitting of atomic coordinates was performed semi-automatically in Chimera [62].

### Modeling an Atomic Structure for IF2

Homology modeling was carried out using the Swiss-Model server [63]. Since for the entire *E. coli* IF2 no homolog of known structure was found, proteins and protein domains with the highest sequence similarity corresponding to domains and sub-domains of IF2 were searched by BLAST [64]. Parts of IF2 were modeled based on the respective homolog structure. Residues 1–50 were modeled using the N-terminal subdomain of IF2 from *E. coli* (pdb code 1ND9; [65]); residues 51–185 with the dynamin-like protein BDLP from *Nostoc punctiforme* (pdb code 2J68; [66]); residues 186–390 with the homologous region of aIF2 from *S. solfataricus* (pdb code 3CW2; [67]); residues 391–559 (corresponding to the G2 domain) and 560–672 (corresponding to the G3 domain) using the IF2/eIF5B from *M. thermautotrophicus* (pdb code 1G7S; [33]); residues 673–779 using the C1-subdomain of IF2 from *B. stearothermophilus* (pdb code 1Z9B; [68]); and residues 780–890 (C2 domain) based on the fMet-tRNA^fMet^-binding domain of IF2 from *B. stearothermophilus* (pdb code 1D1N; [69]). Independent rigid-body fitting of each modeled region into the IF2 density map was performed using Chimera [62]. The initial relative positions of domains was taken as described in the crystal structure for aIF5B [33], taking into account the interaction between domain C2 of IF2 and the initiator tRNA. Connecting residues were adjusted using the Swiss-PDB Viewer software [70]. Molecular dynamics-based flexible fitting of the assembled model was carried out by Flex-EM software [71].

### Accession Numbers

The cryoEM maps for the 30S·mRNA complex (class 1) and for the 30S IC (class 2) have been deposited in the Electron Microscopy Data Bank, http://www.ebi.ac.uk/pdbe/emdb/(accession numbers 1770 and 1771, respectively).

## Supporting Information

Figure S1 SD-ASD helix on cryo-EM maps. Cryo-EM map for class 1 (A) and class 2 (B) after ML3D classification. The maps are rendered semitransparent and fitted with crystal structure of 30S subunit in the complex with mRNA (pdb code: 1JGO; [5]). The SD-ASD helix from the crystallographic structure is shown in blue. Labels: S7 and S11 indicate positions of ribosomal proteins; h, head of the 30S subunit.
(TIF)

Figure S2 Sequence alignment of IF2 from *E. coli* with sequences of proteins used for homology modeling. PDB codes for the different atomic coordinates are indicated under the designation of IF2.
(TIF)

Figure S3 Comparison of the current 30S IC map with previous cryo-EM data from initiation complexes. (A) 30S IC with all three IFs (present work, yellow) aligned with the 30S IC from *T. thermophilus* lacking IF3 (green; [11]). (B) 30S IC and the isolated density for tRNA·IFs extracted from the cryo-EM map of the 70S IC from *E. coli* (blue) [12]. Labels: h, head of the 30S subunit; pt, platform; sp, spur. Arrows point to the junction between fMet-tRNA^fMet^ and IF2.
(TIF)

Figure S4 Visualization of the density attributed to IF3 at different thresholds. In panels (A) and (C) the cryo-EM map is depicted solid; in (B) and (D) the map is semitransparent to show the fitted atomic coordinates for IF3 domains: IF3N (pdb code: 1TIF; [36]) and IF3C (pdb code: 2IFE; [37]). The sigma values used for the rendering are indicated. Thumbnail shows orientation.
(TIF)

Figure S5 Comparison of the 30S conformation from class 1 (30S·mRNA) and class 2 (30S IC), both in semi-transparent renderings, with 30S subunits coming from 70S ribosomes from *E. coli* in rotated (red and solid) and non-rotated (green) states [52]. The conformation of the 30S in the 30S IC is closer to the rotated state. The alignment between density maps was performed by maximum overlapping in the body of the 30S subunits.
(TIF)

## Abbreviations

ASD: anti-Shine-Dalgarno
CTD: C-terminus
FSC: Fourier shell correlation
IF: initiation factor
mRNA: messenger RNA
NTD: N-terminus
SD: Shine-Dalgarno
SRL: sarcin-ricin loop
TIR: translation initiation region
