# Supplementary figures and images for: The Cryo-EM Structure of a Complete 30S Translation Initiation Complex from Escherichia coli
Source: PLoS Biol. 2011 Jul 5;9(7):e1001095. doi: 10.1371/journal.pbio.1001095 (PMC3130014; doi:10.1371/journal.pbio.1001095)

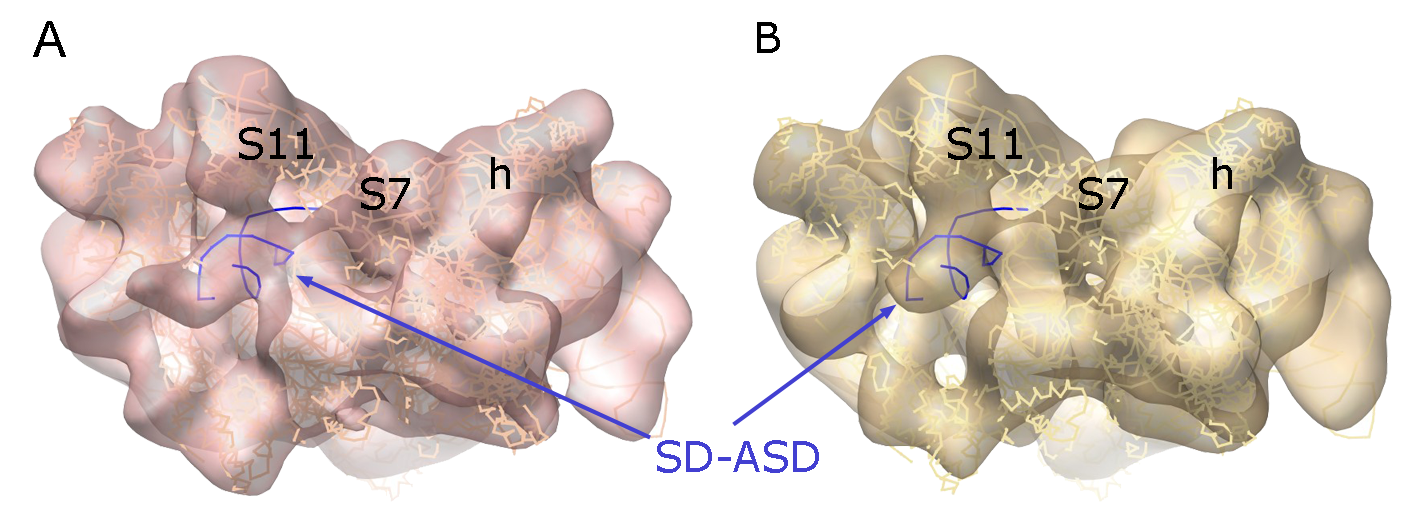

Supplement: Figure S1 — SD-ASD helix on cryo-EM maps. Cryo-EM map for class 1 (A) and class 2 (B) after ML3D classification. The maps are rendered semitransparent and fitted with crystal structure of 30S subunit in the complex with mRNA (pdb code: 1JGO; [5]). The SD-ASD helix from the crystallographic structure is shown in blue. Labels: S7 and S11 indicate positions of ribosomal proteins; h, head of the 30S subunit. (TIF) [file pbio.1001095.s001.tif]

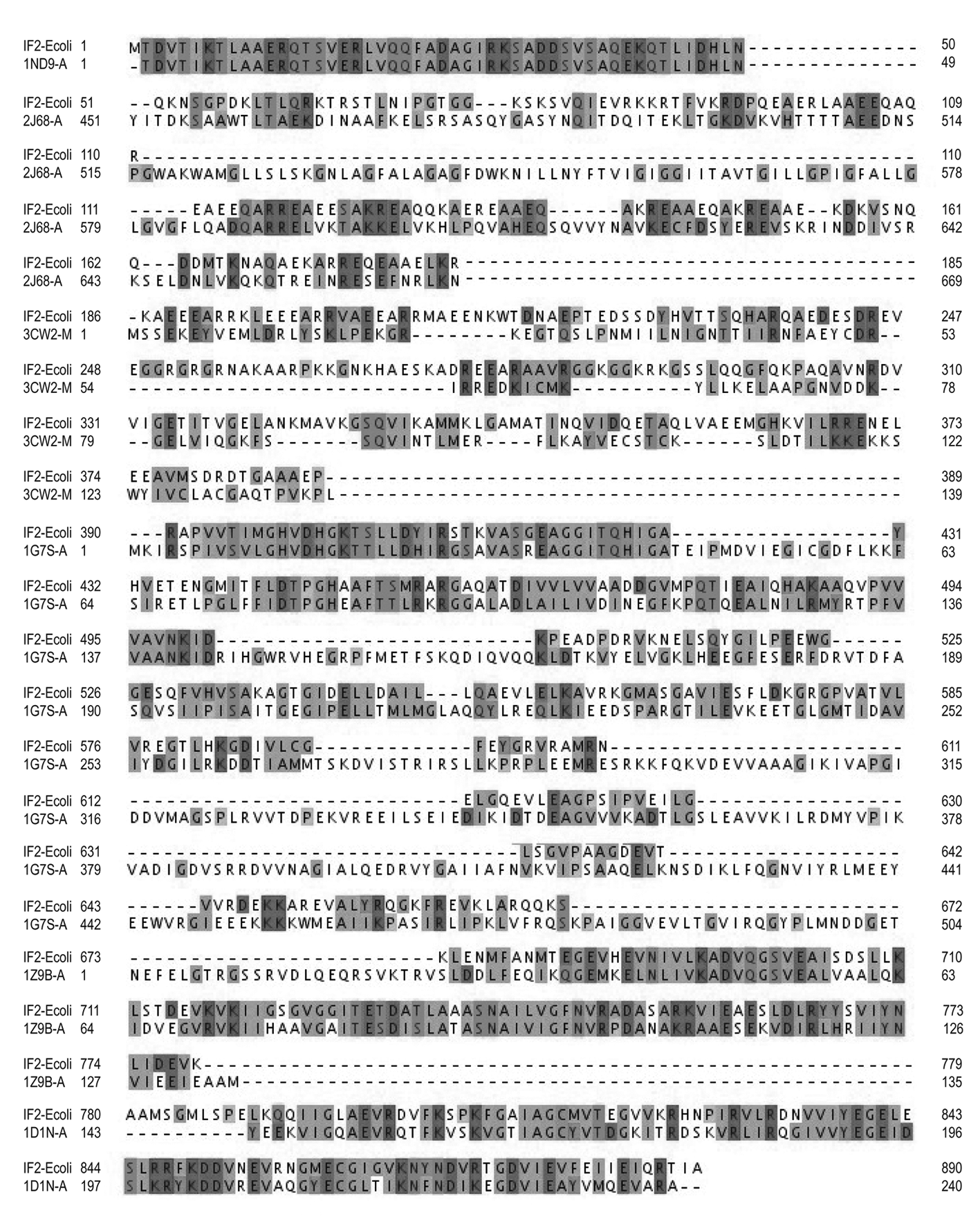

Supplement: Figure S2 — Sequence alignment of IF2 from E. coli with sequences of proteins used for homology modeling. PDB codes for the different atomic coordinates are indicated under the designation of IF2. (TIF) [file pbio.1001095.s002.tif]

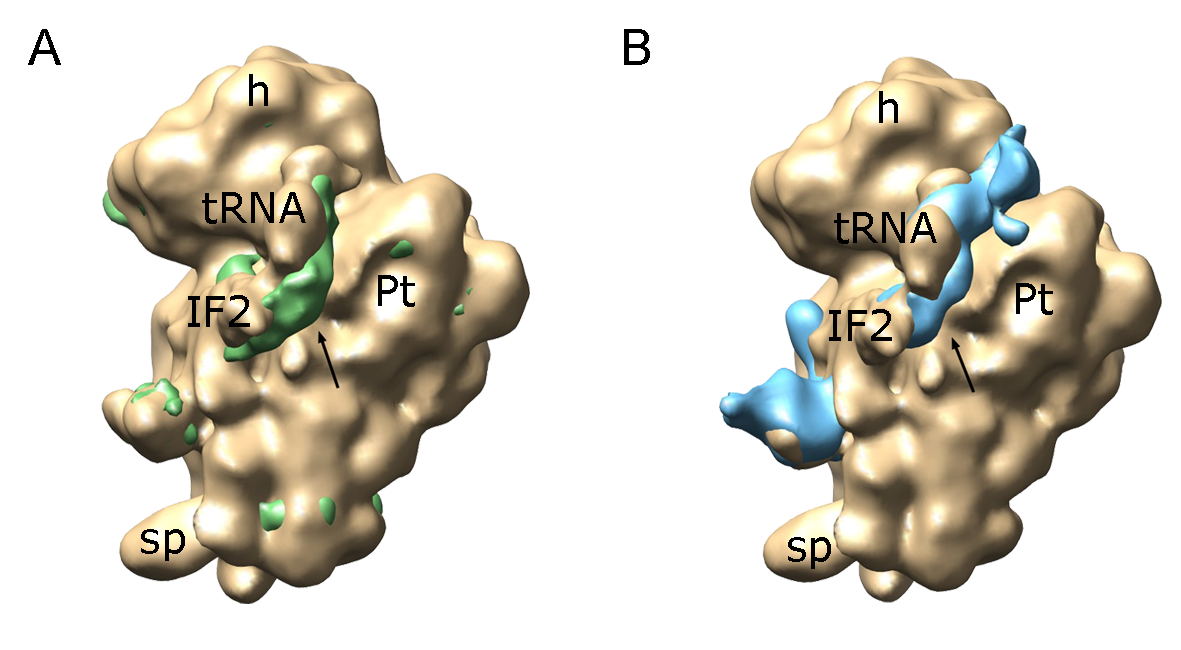

Supplement: Figure S3 — Comparison of the current 30S IC map with previous cryo-EM data from initiation complexes. (A) 30S IC with all three IFs (present work, yellow) aligned with the 30S IC from T. thermophilus lacking IF3 (green; [11]). (B) 30S IC and the isolated density for tRNA·IFs extracted from the cryo-EM map of the 70S IC from E. coli (blue) [12]. Labels: h, head of the 30S subunit; pt, platform; sp, spur. Arrows point to the junction between fMet-tRNAfMet and IF2. (TIF) [file pbio.1001095.s003.tif]

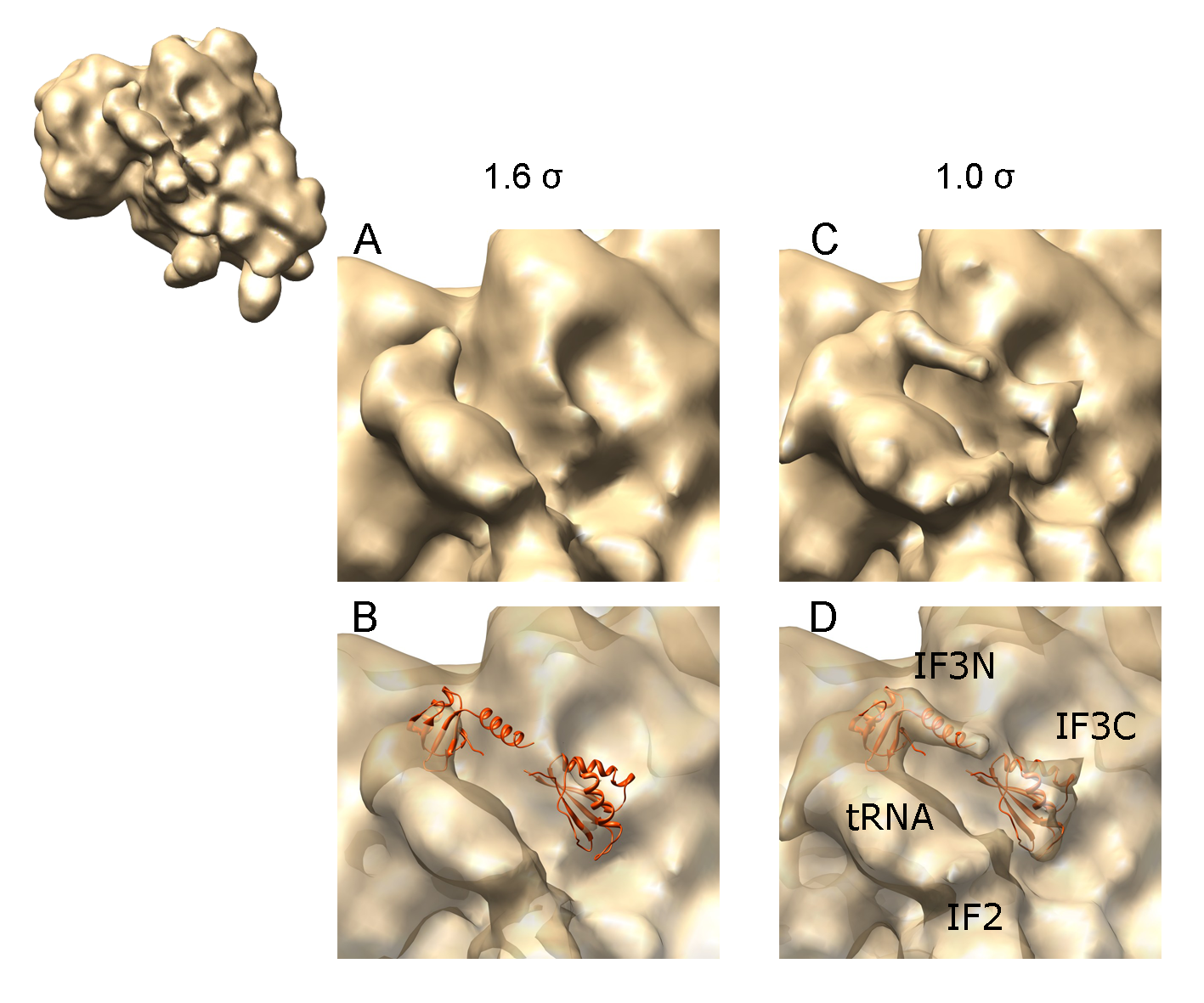

Supplement: Figure S4 — Visualization of the density attributed to IF3 at different thresholds. In panels (A) and (C) the cryo-EM map is depicted solid; in (B) and (D) the map is semitransparent to show the fitted atomic coordinates for IF3 domains: IF3N (pdb code: 1TIF; [36]) and IF3C (pdb code: 2IFE; [37]). The sigma values used for the rendering are indicated. Thumbnail shows orientation. (TIF) [file pbio.1001095.s004.tif]

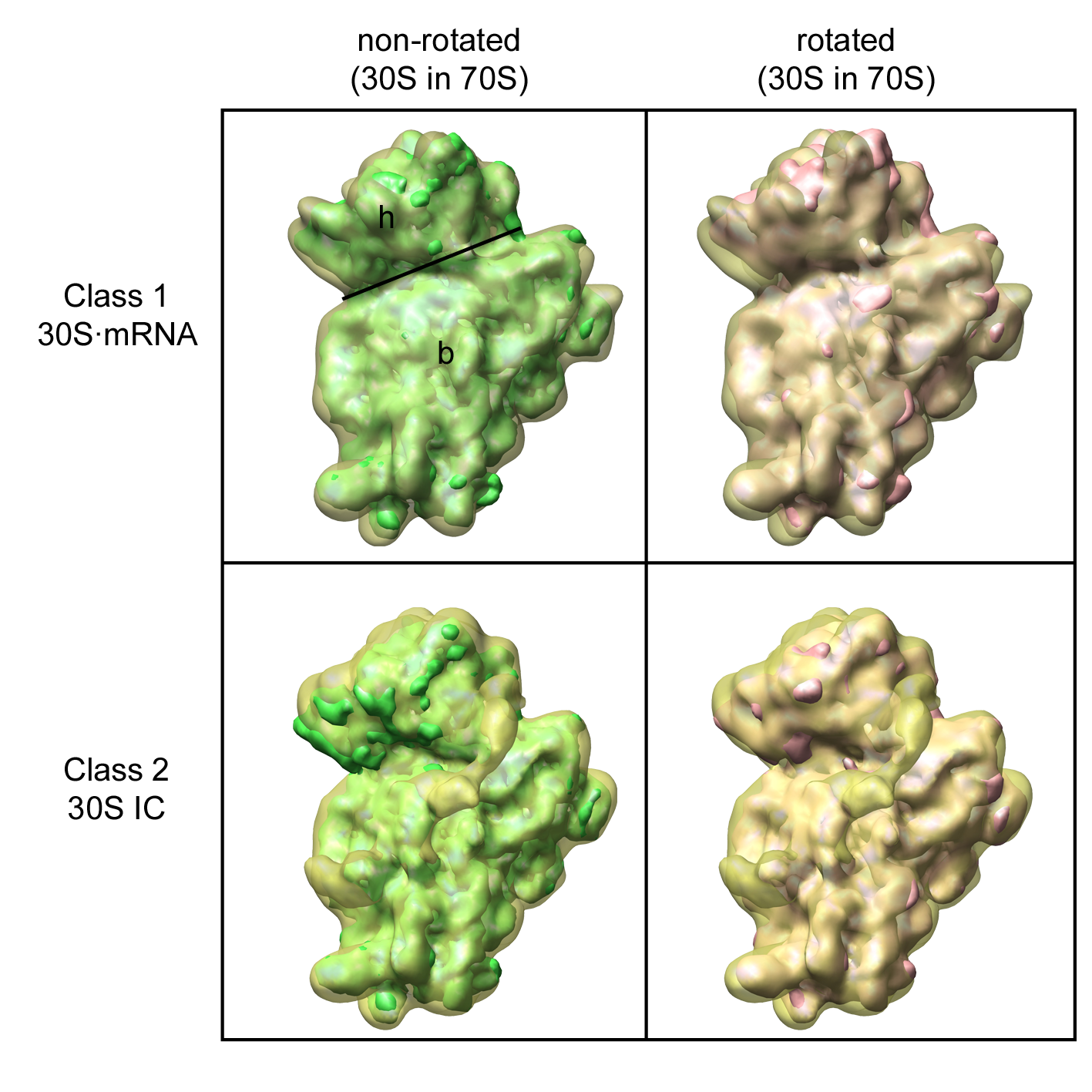

Supplement: Figure S5 — Comparison of the 30S conformation from class 1 (30S·mRNA) and class 2 (30S IC), both in semi-transparent renderings, with 30S subunits coming from 70S ribosomes from E. coli in rotated (red and solid) and non-rotated (green) states [52]. The conformation of the 30S in the 30S IC is closer to the rotated state. The alignment between density maps was performed by maximum overlapping in the body of the 30S subunits. (TIF) [file pbio.1001095.s005.tif]
